# Neurogenesis potential of oligodendrocyte precursor cells from oligospheres and injured spinal cord

**DOI:** 10.3389/fncel.2022.1049562

**Published:** 2022-12-22

**Authors:** Qing Zhao, Yanjing Zhu, Yilong Ren, Shuai Yin, Liqun Yu, Ruiqi Huang, Simin Song, Xiao Hu, Rongrong Zhu, Liming Cheng, Ning Xie

**Affiliations:** ^1^Key Laboratory of Spine and Spinal Cord Injury Repair and Regeneration of Ministry of Education, Orthopaedic Department of Tongji Hospital, School of Medicine, School of Life Sciences and Technology, Tongji University, Shanghai, China; ^2^Division of Spine, Department of Orthopedics, Tongji Hospital, Tongji University School of Medicine, Tongji University, Shanghai, China

**Keywords:** spinal cord injury, oligodendrocyte precursor cell, oligosphere, neurogenesis potential, differentiation, transformation

## Abstract

Severe traumatic spinal cord injury (SCI) leads to long-lasting oligodendrocyte death and extensive demyelination in the lesion area. Oligodendrocyte progenitor cells (OPCs) are the reservoir of new mature oligodendrocytes during damaged myelin regeneration, which also have latent potential for neurogenic regeneration and oligospheres formation. Whether oligospheres derived OPCs can differentiate into neurons and the neurogenesis potential of OPCs after SCI remains unclear. In this study, primary OPCs cultures were used to generate oligospheres and detect the differentiation and neurogenesis potential of oligospheres. *In vivo*, SCI models of juvenile and adult mice were constructed. Combining the single-cell RNA sequencing (scRNA-seq), bulk RNA sequencing (RNA-seq), bioinformatics analysis, immunofluorescence staining, and molecular experiment, we investigated the neurogenesis potential and mechanisms of OPCs *in vitro* and *vivo*. We found that OPCs differentiation and oligodendrocyte morphology were significantly different between brain and spinal cord. Intriguingly, we identify a previously undescribed findings that OPCs were involved in oligospheres formation which could further differentiate into neuron-like cells. We also firstly detected the intermediate states of oligodendrocytes and neurons during oligospheres differentiation. Furthermore, we found that OPCs were significantly activated after SCI. Combining scRNA-seq and bulk RNA-seq data from injured spinal cord, we confirmed the neurogenesis potential of OPCs and the activation of endoplasmic reticulum stress after SCI. Inhibition of endoplasmic reticulum stress could effectively attenuate OPCs death. Additionally, we also found that endoplasmic reticulum may regulate the stemness and differentiation of oligospheres. These findings revealed the neurogenesis potential of OPCs from oligospheres and injured spinal cord, which may provide a new source and a potential target for spinal cord repair.

## 1 Introduction

Oligodendrocytes are critical for myelin formation through repeatedly wrapping neuronal axons (Franklin and Ffrench-Constant, 2008; Irvine and Blakemore, 2008). Severe traumatic spinal cord injury (SCI) leads to locomotor and sensory function loss largely due to long-lasting oligodendrocyte death, extensive demyelination, and Wallerian degeneration (Garcia-Ovejero et al., 2014; Ahuja et al., 2017). Even in moderate SCI and a considerable distance from the lesion area, oligodendrocyte loss and demyelination can be serious (Lytle and Wrathall, 2007). After SCI, the main source of remyelination is from oligodendrocyte progenitor cells (OPCs), also known as NG2 cells. OPCs can migrate, proliferate and gradually differentiate into mature oligodendrocytes and then wrap axons to form a new myelin sheath (Wu et al., 2012).

Previous studies described that OPCs can form oligospheres (Avellana-Adalid et al., 1996; Chen et al., 2007; Pedraza et al., 2008; Gibney and McDermott, 2009; Li et al., 2015). Oligosphere derived OPCs preserve the capacity to express differentiated antigenic and metabolic phenotypes (Avellana-Adalid et al., 1996). When transplanted into the newborn shiverer mouse brain, oligospheres were able to provide a focal reservoir of migrating and myelinating cells (Avellana-Adalid et al., 1996). Recent findings confirmed that OPCs have latent potential for neurogenic regeneration, which may represent a potential target for reprogramming strategies for spinal cord repair (Heinrich et al., 2014; Torper et al., 2015; Tai et al., 2021). However, whether oligospheres derived OPCs can differentiate into neuron-like cells and the neurogenesis potential of OPCs after SCI remains unclear (Chen et al., 2007).

In this study, primary OPCs cultures were used to generate oligospheres and to oberserve the differentiation and neurogenesis potential of oligospheres. We reported a previously undescribed oligospheres derived OPCs could differentiate into neuron-like cells. During the process of differentiation, we firstly discovered the intermediate states of oligodendrocytes and neurons. *In vivo*, by constructing juvenile and adult SCI models, combining the single-cell RNA sequencing (scRNA-seq), RNA sequencing (RNA-seq), bioinformatics analysis, immunofluorescence staining, and molecular experiment, we further investigated the neurogenesis potential of OPCs and its mechanisms after SCI.

## 2 Materials and methods

### 2.1 Primary oligodendrocyte progenitor cells culture

The experimental procedures were designed to minimize the number of animals used as well as animal suffering. All procedures were carried out in accordance with protocols approved by the Institutional Animal Care and Use Committee (IACUC) of the Tongji University School of Medicine. Animals were housed on a 12-h light/dark cycle and had food and water available *ad libitum*. Three or more independent experiments were performed.

Primary OPCs cultures were obtained from postnatal day 1–2 (P1-2) Sprague Dawley rats (Shanghai Jiesijie Laboratory Animal Co., Ltd., China) cerebral cortices and spinal cord. Briefly, cerebral cortices and spinal cord were digested by papain (5 mg/ml, Cat# LS003126, Worthington) and seeded on 0.01% poly-D-lysine (PDL, Cat# C0312, Beyotime Institute of Biotechnology, China)-coated 75-cm^2^ flasks. Dissociated cells were maintained in DMEM-F12 (Cat# 11330057, Gibco) containing 10% fetal bovine serum (FBS, Cat# 16000-044, Gibco) and 1% penicillin/streptomycin (Cat# 15140122, Gibco) at 37°C for 7 days. During this time, primary OPCs cultures were used for immunofluorescence staining to detect the differences in OPCs differentiation between brain and spinal cord.

Adherent cells were dissociated by Accutase (Cat# A1110501, Gibco) after reaching 70–80% confluency for 10 min at 37°C and then reseeded in 100 mm culture dishes (Cat# 430167, Corning) for 1 h. Then, the supernatant fluid was collected to remove the astrocytes and neuron. As described in a previous study (Hattori et al., 2017; Miyamoto et al., 2020), the medium was replated onto PDL-coated plates in OPCs proliferation medium to only collect OPCs, containing neurobasal medium (Cat# 10888022, Gibco), 10 ng/ml PDGF-AA (Cat# 315-17, Peprotech), 10 ng/ml bFGF (Cat# 45033, Peprotech), 2 mM glutamine (Cat# G7513, Sigma), 1% penicillin/streptomycin, 5 ng/ml insulin (Cat# P3376-100 IU, Beyotime Institute of Biotechnology, China), 20 ug/ml NT3 (Cat# 450-03-10, Peprotech), and 2% B27 supplement (Cat# A3582801, Gibco). Finally, OPCs with >95% purity were obtained (by cell count) and reseeded in 100 mm culture dishes (Cat# 430167, Corning) and poly-D-lysine-coated 12-well culture plates with cell climbing sheets for the next experiment.

### 2.2 Primary OPCs culture to generate oligospheres

Primary OPCs were obtained as described above. When OPCs with >95% purity were obtained, dissociated cells were reseeded in a new 100 mm non-coated Corning dish with OPCs proliferation medium. Oligospheres started to form after 4–5 days of culture, during which time the oligospheres were blown up and dissociated for resuspension, and facilitated oligospheres formation. The culture medium was changed every 3 days depending upon the cellular density. Approximately every 3 days half of the media was changed and cells passaged without digestive enzyme digestion. After elimination of adhering cells, oligospheres were centrifuged (800 rpm for 5 min) and resuspended in OPCs proliferation medium.

When oligospheres reached the fourth generation (F4), they were seeded into glass coverslips with PDL-coated 12-well culture plates. Immunofluorescence staining was performed to detect the differentiation of oligospheres at 4 h (Day 0) and Days 1, 3, 6, 9, 14, 21, and 28 after plating. We also found that some oligospheres tended to attach to the bottom of the plate, as previously reported (Chen et al., 2007), and free-floating oligospheres only were used for passaging. The oligosphere images under white light were taken by an Olympus microscope. Three independent experiments were performed.

### 2.3 Oxygen-glucose deprivation model and inhibitors treatment

To mimic hypoxic-ischaemic injury, an oxygen-glucose deprivation (OGD) model (O_2_ < 0.1%) was constructed by AnaeroPack (Cat# D07, Mitsubishi Gas Chemical Company, Japan) in a 2.5 L closed plastic box. After reaching 70–80% confluency at 3 days, OPCs were incubated in hypoxic conditions with glucose-free DMEM (Cat# 11966025, Gibco) for 3 h and then switched to DMEM-F12 containing 10% FBS and 1% penicillin/streptomycin. The inositol-requiring protein 1α (IRE1α) inhibitor STF083010 (Cat# 307543-71-1, MedChemExpress) (Papandreou et al., 2011; Zhao et al., 2017a) and protein kinase RNA-like ER kinase (PERK) inhibitor GSK2656157 (Cat# 1337532-29-2, MedChemExpress) (Atkins et al., 2013) with different concentrations were used to treat primary oligodendrocytes at 1 h before and after OGD stimulation, respectively. Then, OPCs were collected for quantitative real-time PCR (RT–PCR), and live/dead cells by Calcein AM/PI double staining and flow cytometry of apoptosis by Annexin V-EGFP/PI double staining (Cat# KGA103, Jiangsu KeyGen BioTech Corp., Ltd. China). Staining methods followed the reference specification. Three independent experiments were performed.

### 2.4 Spinal cord contusion model construction

Eight-week-old wild-type C57BL/6 J female mice (Shanghai Jiesijie Laboratory Animal Co., Ltd.) were used for contusion model construction, including sham group (*n* = 9) and SCI group (*n* = 9). Spinal cord contusion models at T10 were established using the MASCIS Impactor Model III (W.M. Keck Center for Collaborative Neuroscience, Rutgers, The State University of New Jersey, USA) (Duan et al., 2021).

Briefly, mice were weighed and deeply anesthetized with iso-flurane evaporated in a gas mixture containing 70% N_2_O and 30% O_2_ through a nose mask. The back skin was shaved and cleaned with 75% alcohol. A laminectomy at T10 was performed to remove the part of the vertebra overlying the spinal cord, exposing a circle of dura through an operating microscope (Zeiss, Germany) and rodent stereotaxic apparatus (RWD Life Science Co., Ltd., Shenzhen, China). The spinal column was stabilized using lateral clamps over the lateral processes at T9 and T11. Contusion was performed at T10 with a 5 g impactor and 6.25 mm height with a force of about 60kdyn, which could cause a moderate injury as previously reported (Wu et al., 2013, 2014), and then the wound was sutured. Sham mice underwent laminectomy but not contusion.

The following symbols were indicators of a successful contusion model: (1) the impact point was located in the middle of T10, (2) paralysis of both hindlimbs occurred after awakening. Unsuccessful models were excluded in the following experiment and analysis. Mice had received natural illumination to keep warm before and after the surgery. Urine was manually expressed from the bladders of the injured mice twice per day until autonomous urination recovered. Six weeks after contusion, the mice were sacrificed for immunofluorescence staining.

### 2.5 Spinal cord crash model construction

Eight-week-old wild-type C57BL/6 J female mice (adult mice) and 1-week-old and 2-week-old juvenile mice were used for spinal cord crash model construction, including sham and SCI groups of 1-week-old mice (Sham_1 W, *n* = 3; SCI_1 W, *n* = 3); sham and SCI groups of 2-week-old mice (Sham_2 W, *n* = 3; SCI_2 W, *n* = 3); sham and SCI groups of 8-week-old mice (adult female mice, Sham_8 W, *n* = 3; SCI_8 W, *n* = 3). Three biological repeats were performed.

The pre-operative disinfection, anesthesia, and post-operative care were conducted as the same as contusion model. The crash model was also performed at T10 with No. 5 Dumont forceps fixed on a stereotaxic apparatus and persisted for 3 s. The paralysis of both hindlimbs occurred after awakening were indicators of a successful crash model. Unsuccessful models were excluded in the following experiment and analysis. Sham mice received laminectomy without crash. The juvenile mice were returned to the female mice cage to continue feeding after crash. Two weeks after crash, the mice were sacrificed for RNA-sequencing.

### 2.6 Immunofluorescence staining and analysis

Briefly, mice were under deep anesthesia and intracardially perfused with saline and then with 4% paraformaldehyde at the indicated time. After post-fixation and cryoprotection, the dissected 6 mm segment of spinal cord centered at the lesion area, or 2 mm segment of lumbar enlargement (L4-5), was coronally sectioned at 12 μm thickness and thaw-mounted onto Superfrost Plus slides (Citotest Labware Manufacturing Co., Ltd., Haimen, China). Spinal cord sections were prepared for immunofluorescence staining following procedures described previously (Ren et al., 2017). The culture cells immunofluorescence staining of methods have been described previously (Zhu et al., 2021).

The primary antibodies used were as follows: glial fibrillary acidic protein (GFAP, Cat# ab4674, Abcam, 1:500), myelin basic protein (MBP, Cat# MAB386, Millipore, 1:500), oligodendrocyte transcription Factor 2 (Oligo2, Cat# AF2418, R&D system, 1:500), neural/glial antigen 2 (NG2, Cat# AB5320, Millipore, 1:500), Nestin (Cat# ab134017, Abcam, 1:500), MAP2 (Cat# ab32454, Abcam, 1:500), Tau (Cat# MAB3420, Millipore, 1:500); the secondary antibodies were Alexa^®^ Fluor 488 (Cat# A-32814, Invitrogen, 1:500), Alexa^®^ Fluor 488 (Cat# A-32790, Invitrogen, 1:500), Alexa^®^ Fluor 555 (Cat# A-32773, Invitrogen, 1:500), Alexa Fluor^®^ 594 (Cat# ab150156, Abcam, 1:500), Alexa^®^ Fluor 594 (Cat# ab150176, Abcam, 1:500), Alexa^®^ Fluor 647 (Cat# A-32795, Invitrogen, 1:500). Fluorescent antibodies of the same channel including Alexa^®^ Fluor 488 and Alexa^®^ Fluor 594, were used to match species differences during immunofluorescence staining. The nuclei were stained with 4′,6-diamidino-2-phenylindole (DAPI, Cat# C1002, Beyotime Institute of Biotechnology), and fluorescence images were taken using confocal microscopy (LSM 700, Carl Zeiss, Jena, Germany). Image acquisition was performed with ZEN 2.3 (blue edition, Carl Zeiss), and micrographs were assembled using Adobe Illustrator CC 2018.

For immunofluorescence analysis, lesion area covered the lesion core, rostral and caudal injured spinal cords with a distance of 500 μm from lesion core, respectively. A total of three sections (eight areas of rostral and eight of caudal injured spinal cords per section) were randomly selected in per mice. Then the 160 μm × 160 μm images were exported by ZEN 2.3. For culture cells immunofluorescence staining, the images were directly exported by ZEN 2.3. Image-J software with customized macros was used to quantify the fluorescence intensity of proteins including the MBP, Olig2, and NG2 in the images mentioned above. For sham group and treatment groups, three mice per group were tested. For culture cells, three biological repeats were conducted.

### 2.7 RNA-sequencing and bioinformatic analysis

RNA-seq was used to examine the transcriptomes of the lesion site at 2 weeks post injury (WPI), including sham and SCI groups of 1-week-old mice (Sham_1 W, *n* = 3; SCI_1 W, *n* = 3, crash model); sham and SCI groups of 2-week-old mice (Sham_2 W, *n* = 3; SCI_2 W, *n* = 3, crash model); sham and SCI groups of 8-week-old mice (adult female mice, Sham_8 W, *n* = 3; SCI_8 W, *n* = 3, crash model). Total RNA from 18 samples with three biological replicates for each group was quantified to obtain RNA data.

Briefly, animals were euthanized after terminal anesthesia by pentobarbital overdose. Then, spinal cord tissue was dissected from the lesion site under a dissecting microscope, and 2 mm segments of the lesion site were harvested and marked. The tissues were sent to the Beijing Genomics Institute (BGI) Company (Shenzhen, China) in solid carbon dioxide for further RNA-seq. The sequencing was performed on a DNBSEQ system at BGI Company. Total RNA was isolated, and RNA sequencing libraries were generated. Bioinformatics analysis was carried out with the online platform Dr. Tom (BGI Company). Only genes with transcripts per million (TPM) > 1 were analysed. Differentially expressed genes (DEGs) were identified using the DEGseq2 method and screened with the criteria of q value ≤ 0.05 and log2FC ≥ 0.6, where FC represents the fold change. The sequence and sample data have been deposited in the NCBI database under Sequence Read Archive (SRA) with Bioproject identification number PRJNA847738 (Accession number: SRR19612226–SRR19612243).

To further detect oligodendrocyte activation in lumbar enlargement (L4-5), RNA-seq was used to examine the transcriptomes of lumbar enlargement tissues at 6 WPI, including the sham (*n* = 6, female) and SCI groups (*n* = 6, female, contusion model). Total RNA from 12 samples with six biological replicates for each group was quantified to obtain RNA data. Spinal cord tissue was dissected from lumbar enlargements (L4-5) under a dissecting microscope, and 2 mm segments were harvested and marked. RNA-seq and bioinformatic analysis were also performed on a DNBSEQ platform and the online platform Dr. Tom (BGI Company). Only genes with TPM > 1 were analyzed. DEGs were identified using the DEGseq method and screened with the criteria of q value ≤ 0.05 and log2FC ≥ 0.6. The sequence and sample data have been deposited in the NCBI database under Sequence Read Archive (SRA) with Bioproject identification number PRJNA847704 (Accession number: SRR19611667–SRR19611678).

To further detect oligodendrocyte activation at different time after SCI, the series matrix file data of GSE45006 were downloaded from the Gene Expression Omnibus (GEO) public database, and the annotation platform was GPL1335. Data from 24 samples from the lesion area of the injured spinal cord with complete expression profiles were extracted. Bioinformatics analysis was also carried out with the online platform Dr. Tom (BGI Company). DEGs were identified using the DEGseq method and screened with the criteria of *p*-value ≤ 0.01 and log2FC ≥ 2. The scRNA-seq results were analyzed through online data from injured mouse spinal cords^1^ (Milich et al., 2021).

### 2.8 Quantitative real-time PCR

The total RNA of cells was isolated with RNAiso plus (Cat# 9108, Takara). The concentration and purity of RNA samples were measured using a Nanodrop ND-2000 (Thermo Science, MA, USA) for further experiments. Five hundred nanograms of RNA was converted to complementary DNA (cDNA), which was synthesized with a PrimeScript reverse transcriptase kit (Cat# RR037A, Takara). RT–PCR was performed using a TB Green TM Premix Ex Taq Kit (Cat# RR820A, Takara) on a Light Cycler Real-Time PCR System (480II, Roche). The primer sequences (Shanghai Generay Biotech Co., Ltd., Shanghai, China) were designed through Primer-BLAST^2^ and are listed in Supplementary Table 1. The relative amounts of mRNA were calculated using the ΔΔCt relative quantification method. GAPDH served as the control gene, and the mRNA levels of specific genes were normalized to GAPDH. Calculations and statistics were performed in Microsoft Excel version 16.36. Graphs were plotted in GraphPad Prism 8 version 8.4.3. Three biological repeats were performed.

### 2.9 Statistical analysis

All experiments were conducted with three or more duplicates. All continuous data were shown as mean ± SEM. One-way ANOVA was performed followed by Student Newman–Keuls *post-hoc* test for continuous data. *P*-values < 0.05 were considered statistically significant. Data analyses were conducted using the Statistical Analysis System (SAS), version 9.4 (SAS Institute, Inc., Cary, NC, USA). Plots were generated using GraphPad Prism 8 software (GraphPad Software, San Diego, CA, USA).

## 3 Results

### 3.1 OPCs differentiation were different between brain and spinal cord

**FIGURE 1:**
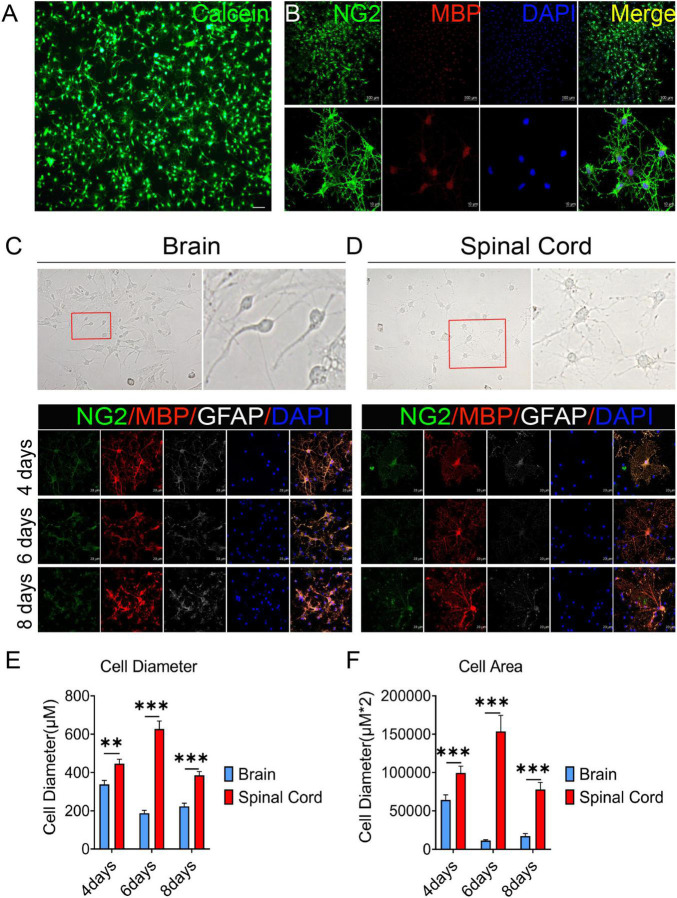
The oligodendrocyte progenitor cells (OPCs) differentiation and oligodendrocytes morphology are different between the brain and spinal cord. **(A,B)** OPCs with >95% purity were obtained (by cell count) and identified by Calcein AM staining and the expression of oligodendrocyte lineage cell markers NG2 (green) and MBP (red). **(C,D)** Spinal cord-derived OPCs were easier to differentiate and mature than brain-derived cells. Immunofluorescence staining of NG2 (green), MBP (red), and GFAP (white) was used to detect the dynamic changes in oligodendrocytes. *n* = 3 biological repeats. Scale bars are indicated in the pictures. **(E,F)** The histograms showed the statistical results of oligodendrocytes diameter and area. *n* = 3 biological repeats. Values are the mean ± SEM. Statistical significance was determined by one-way ANOVA followed by Student Newman–Keuls *post-hoc* test. ^**^P < 0.01, ^***^P < 0.001.

In this study, OPCs with >95% purity were obtained (by cell count) and identified by Calcein AM staining and the expression of oligodendrocyte lineage cell markers NG2 and MBP (Figures 1A, B). Previous reports have revealed that oligodendrocytes are heterogeneous (Rowitch and Kriegstein, 2010; Bechler et al., 2015). We also observed that there were obvious differences in OPCs differentiation between brain and spinal cord (Figures 1C, D). On the third day of primary cell culture (mixed with OPCs, astrocytes, etc.) in the medium of DMEM-F12 containing 10% FBS and 1% penicillin/streptomycin, we found brain-derived cells were mainly OPCs with a dark cell body, tadpole-like, and only 2–3 branches (Figure 1C). In contrast, spinal cord-derived OPCs were easier to differentiate than brain-derived, reflected by the significant increase in branches and processes (Figure 1D). Immunofluorescence staining of NG2, MBP, and GFAP was further used to detect dynamic changes of OPCs in both differentiation and morphology (Figures 1C, D). We delineated cell morphology, and calculated the diameter and area of OPCs differentiated oligodendrocytes (Supplementary Figure 1). When compared with brain, we found that spinal cord-derived oligodendrocytes had larger cell areas and longer diameters after maturation (Figures 1C–F). Spinal cord-derived oligodendrocytes had showed longer diameter and larger cell area at the 4th day, peaked at the 6th day and were maintained until the 8th day (Figures 1E, F). Thus, there were significant differences in OPCs differentiation and oligodendrocytes morphology between the brain and spinal cord.

### 3.2 OPCs were involved in oligospheres formation *in vitro*

**FIGURE 2:**
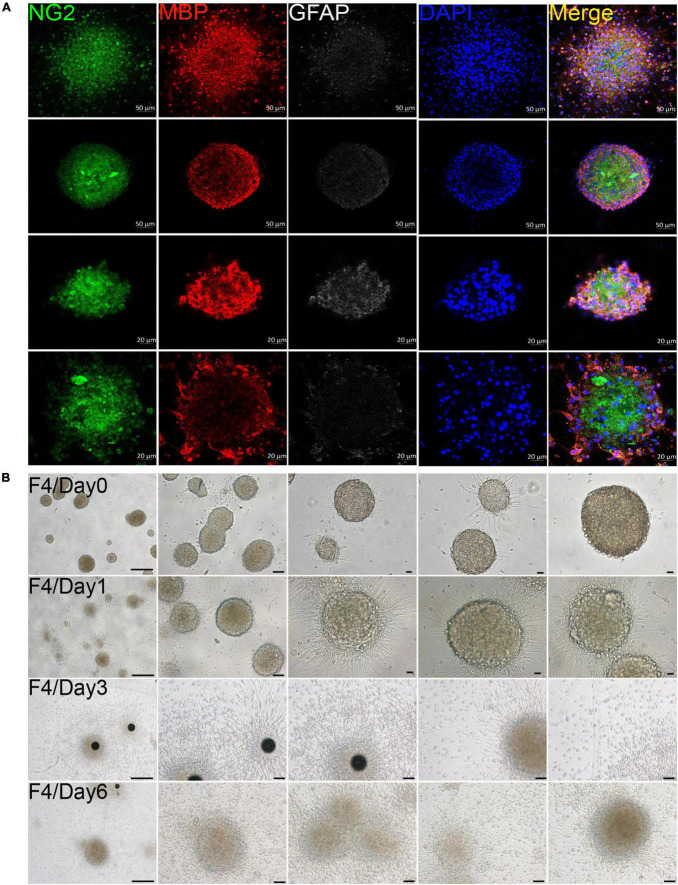
Oligodendrocyte progenitor cells (OPCs) were involved in oligospheres formation. **(A)** Oligodendrocytes from brain formed into oligospheres on the 8th day, as observed NG2 (green), MBP (red), and GFAP (white). *n* = 3 biological repeats. Scale bars are indicated in the pictures. **(B)** Oligospheres from F4 generation were plated on PDL-coated 12-well plates to detect the differentiation at different time. F4 = the fourth generation. *n* = 3 biological repeats. Scale bars are indicated in the pictures.

In our culture system, we found that OPCs from both brain and spinal cord could gather into oligospheres on the 8th day of OPCs proliferation (Figure 2A and Supplementary Figure 2). Compared with spinal cord, brain-derived oligospheres were more obvious in spherical and oval shapes (Figure 2A and Supplementary Figure 2). Thus, we mainly focused on the formation of brain-derived oligospheres in the next experiment and analysis. Oligospheres were plated on PDL-coated coverslips in OPCs proliferation medium to detect their differentiation (Figure 2B and Supplementary Figures 3A–C). We found that the volume of oligospheres in the first generation (F1) was small (Supplementary Figure 3A). Oligospheres gradually differentiated after plating (Supplementary Figure 3A). As previously reported (Chen et al., 2007), cells from oligospheres migrated away to form individual OPCs with bipolar and tripolar morphologies (Supplementary Figure 3A). According to cell morphology, we found that a large number of OPCs and oligodendrocytes were formed and migrated out of the oligospheres at different time in the F1 generation (Supplementary Figure 3A). When oligospheres reached the F2 generation, the size and diameter of oligospheres increased with round and oval shapes (Supplementary Figure 3B). In the F3 generation, the oligospheres reached a larger size and diameter (Supplementary Figure 3C). In the F4 generation, the size and diameter of the oligospheres reached the most significant values (Figure 2B), which could also differentiate into a large number of OPCs and oligodendrocytes at different time (Figure 2B). We also found that a number of astrocytes were migrated out of the oligospheres at different time (Figure 2B and Supplementary Figure 3) as previous study reported (Chen et al., 2007).

### 3.3 Oligospheres can differentiate into oligodendrocytes and neuron-like cells

**FIGURE 3:**
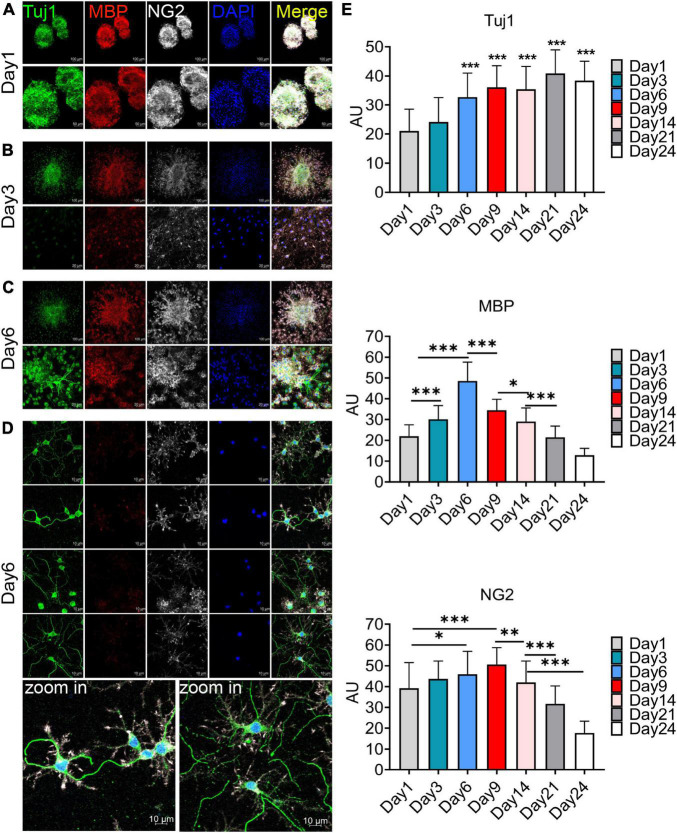
Immunofluorescence staining to detect the cellular composition of oligospheres at different differentiation period. **(A–D)** F4 generation oligospheres were used for immunofluorescence staining to detect the cellular composition of oligospheres at different time with Tuj1 (green), MBP (red), and NG2 (white). *n* = 3 biological repeats. Scale bars are indicated in the pictures. **(E)** The histograms showed the statistical results of fluorescence intensity. Values are the mean ± SEM. Statistical significance was determined by one-way ANOVA followed by Student Newman–Keuls *post-hoc* test. *P < 0.05, ^**^P < 0.01, ^***^P < 0.001. *n* = 3 biological repeats. Scale bars had been indicated in pictures.

The F4 generation oligospheres were used to observe differentiation at different time through immunofluorescence staining with markers of oligodendrocytes (MBP), OPCs (NG2), and neurons (Tuj1). On the first day after plating, there were a large number of TuJ1, MBP, and NG2 positive cells in the oligospheres (Figure 3A). Nearly all oligospheres were Tuj1 + MBP + NG2 + triple positive oligospheres (Figure 3A). Meanwhile, MBP + positive oligodendrocytes and NG2 + OPCs were wrapped by Tuj1 positive neuronal cells (Figure 3A). As expected, we found almost all oligospheres were Nestin positive (Supplementary Figures 4A, B), which indicated that oligospheres have neural stemness. On the third day after plating, the oligospheres differentiated into a majority of MBP + positive oligodendrocytes, NG2 + OPCs, and a small number of Tuj1 positive neurons (Figure 3B).

A previous study reported that no neuronal cells indicated by the expression of Tuj1 were detected in an isolated OPCs population, although approximately 2% of cells represented other neuroglial cells or early progenitors, as evidenced by the expression of Nestin and GFAP (Chen et al., 2007). Interestingly, we found a large number of Tuj1 + MBP + NG2 + triple-positive oligodendrocytes on the sixth day after plating (Figure 3C), among which a large number of these cells had Tuj1-positive neurite-like branches (Figure 3D). We speculated that these cells may be in the intermediate state of oligodendrocytes and neurons during oligospheres differentiation, which indicated that these oligodendrocytes may *trans*-differentiate into neurons in this culture system. In addition, we detected the differentiation of oligospheres for a longer time. On the 9th, 14th, 21st, and 28th days of oligospheres differentiation, an increasing number of Tuj1 positive cells were found at different time (Supplementary Figures 5A–D). Through statistical fluorescence intensity at different differentiation time, we found that Tuj1 expression gradually increased, while NG2 and MBP decreased (Figure 3E). These results reflect that oligospheres differentiating into oligodendrocytes gradually decreased, which indicated that OPCs may *trans*-differentiate into neuron-like cells. However, these cells not grew long axon-like branches (Figure 3D and Supplementary Figures 5A–D). For most astrocytes were removed in our culture system, we speculate that one of the reasons may be the lack of secreted astrocyte factors, including BDNF and GDNF, to promote and guide the growth of neurons (Cunningham and Su, 2002; Degos et al., 2013).

Furthermore, we used more neuron markers for immunofluorescence staining, including MAP2 and Tau. The effects of different culture mediums on the results were also observed. Firstly, we found that on the 6th day of oligospheres differentiation, a large number of cells expressed MAP2 in proliferation medium (Supplementary Figure 6A). The cells had few branches and were in the immature state (Supplementary Figure 6A). In contrast, in DMEM-F12 with 5% FBS medium and on the 6th day of oligospheres differentiation, we found that a large number of cells also expressed MAP2. However, the cells were relatively mature and with abundant branches (Supplementary Figure 6B). Secondly, under the condition of proliferation medium culture, and on the 6th day of oligospheres differentiation, we found that some NG2 positive cells (OPCs) expressed Tau protein, and the branches were long (Supplementary Figure 6C). However, in DMEM-F12 with 5% FBS medium and on the 6th day of oligospheres differentiation, NG2 positive cells (OPCs) were few, and almost no NG2 positive cells (OPCs) expressed Tau protein (Supplementary Figure 6D). Thus, these results indicated that oligospheres can differentiate into neuron-like cells only in the proliferation medium.

### 3.4 OPCs are activated in the lesion area and lumbar enlargement after SCI

**FIGURE 4:**
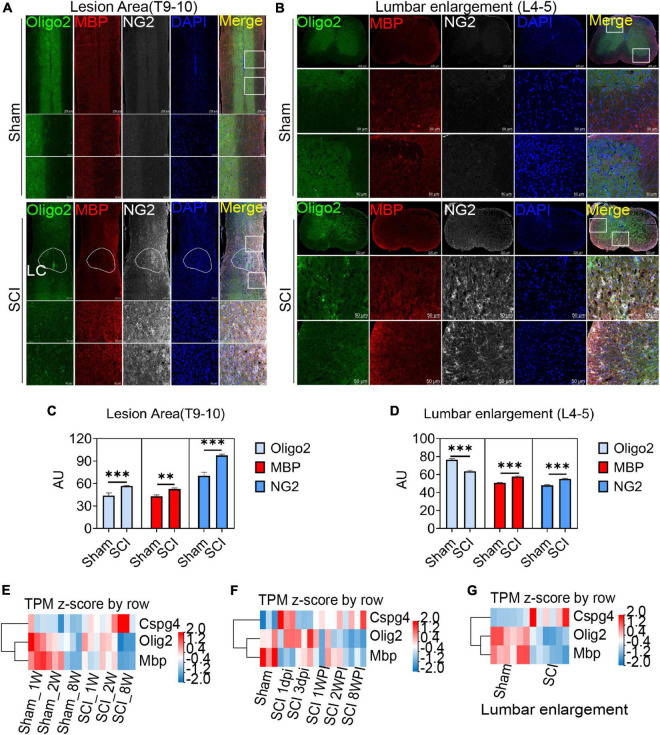
Oligodendrocyte progenitor cells (OPCs) are activated in the lesion area and lumbar enlargement after SCI. **(A,B)** Immunofluorescence staining to detect the expression of oligodendrocyte lineage cell markers in the lesion area and lumbar enlargement after SCI. Olgio2 (green), MBP (red), and NG2 (white). *n* = 3 biological repeats. Scale bars are indicated in the pictures. LC = lesion core. **(C,D)** The histogram and statistical results of Oligo2, MBP, and NG2 fluorescence intensity. *n* = 3 biological repeats. Values are the mean ± SEM. Statistical significance was determined by one-way ANOVA followed by Student Newman–Keuls *post-hoc* test. ^**^P < 0.01, ^***^P < 0.001. **(E–G)** RNA-seq results showed transcript changes in oligodendrocyte lineage cells both in the lesion area and lumbar enlargement at different ages and time. dpi, day post-injury; WPI, week post-injury; 1 W, 1-week-old; 2 W, 2-week-old; 8 W, 8-week-old; sham_1 W, 1-week-old sham group; sham_2 W, 2-week-old sham group; sham_8 W, 8-week-old sham group; SCI_1 W, 1-week-old SCI group; SCI_2 W, 2-week-old SCI group; SCI_8 W, 8-week-old SCI group.

After spinal cord contusion injury, oligodendrocytes in the lesion area were activated at 6 weeks post injury (WPI) when compared with the sham group, reflected by the expression of the oligodendrocyte marker Oligo2, myelin marker MBP, and OPCs marker NG2 (Figures 4A, C). The demyelination of the lesion core was significant and not effectively repaired (Figure 4A). Meanwhile, the activation of OPCs (NG2+) in the lumbar enlargement was still very significant even at a distance from the injured area (Figures 4B, D).

Through RNA-seq, we further observed transcriptional changes in oligodendrocyte lineage cells both in the lesion area and lumbar enlargement at different ages and time (Figures 4E–G). Compared with juvenile mice, we confirmed that the transcripts of MBP and Oligo2 in adult mice decreased significantly at 2 WPI (Figure 4E), which indicated that a majority of oligodendrocyte death and extensive myelin sheath loss occurred in the lesion area. Meanwhile, NG2 (CSPG4) transcripts in adult mice were significantly up-regulated when compared with juvenile mice but did not promote remyelination of the lesion core (Figure 4E). Additionally, we also found that the transcripts of MBP and Oligo2 decreased with time after SCI, while the transcripts of NG2 were gradually up-regulated but did not promote the regeneration of the myelin sheath (Figure 4F). Interestingly, the transcripts of NG2 were also significantly up-regulated in lumbar enlargement, and the transcripts of MBP and Oligo2 were down-regulated even at a distance from the injured area at 6 WPI (Figure 4G).

### 3.5 Combining scRNA-seq and RNA-seq data to investigate the neurogenesis potential of OPCs after SCI

**FIGURE 5:**
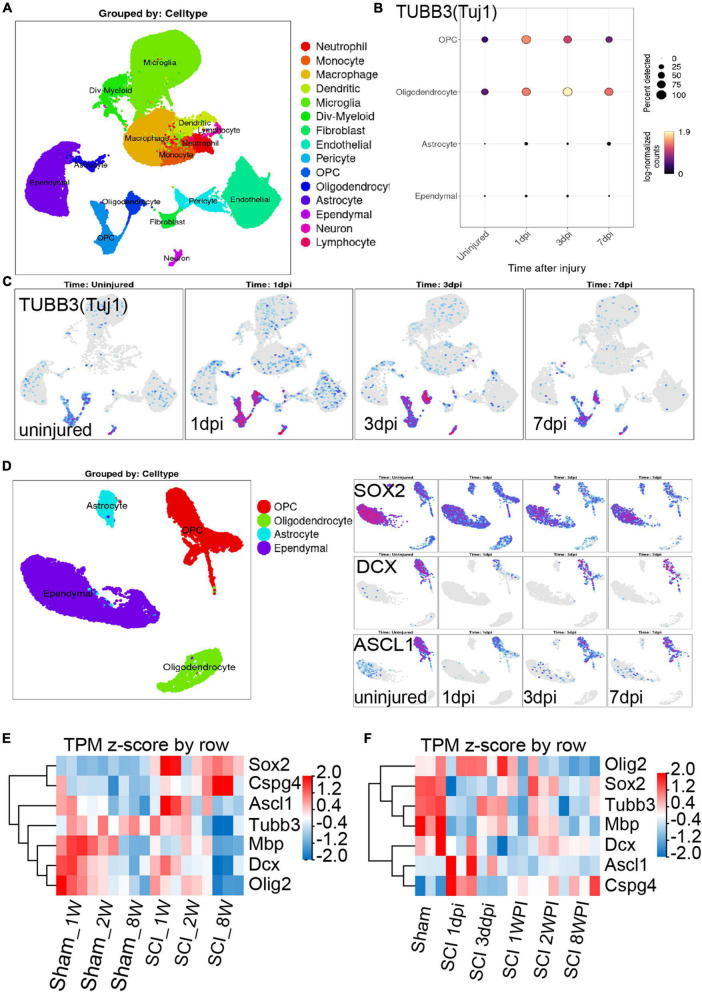
Combining the scRNA-seq and RNA-seq data to investigate the neurogenesis potential of oligodendrocyte progenitor cells (OPCs) after spinal cord injury (SCI). **(A–C)** scRNA-seq results show that oligodendrocyte lineage cell along with neurons were Tuj1-specific expressing cells under normal conditions and after SCI. dpi = day post-injury. **(D)** Sox2, DCX, and ASCL1 were highly or specifically expressed in OPCs under normal conditions, while these factors were significantly down-regulated after SCI. dpi = day post-injury. **(E,F)** RNA-seq results show the transcript changes of the above factors along with the marker of oligodendrocyte cell lines in the lesion area at different ages and time. dpi, day post-injury; WPI, week post-injury; 1 W, 1-week-old; 2 W, 2-week-old; 8 W, 8-week-old; sham_1 W, 1-week-old sham group; sham_2 W, 2-week-old sham group; sham_8 W, 8-week-old sham group; SCI_1 W, 1-week-old SCI group; SCI_2 W, 2-week-old SCI group; SCI_8 W, 8-week-old SCI group.

There is a dynamic process of pathophysiological changes after SCI. Its pathophysiology comprises acute and chronic stages and incorporates a cascade of pathogenic mechanisms (cell death, excitotoxicty, inflammation, neurodegneraton, demyelination, remielination, scar formation, etc.) (Anjum et al., 2020). The regional microenvironment of injured spinal cord is quite different from that of cells cultured *in vitro*. *In vitro* experiments are also difficult to simulate complex environmental changes *in vivo*. Thus, in order to know more about the dynamic changes in different periods after SCI *in vivo*, we analysis the RNA-seq and online scRNA-seq data. By analyzing online scRNA-seq data from the injured mouse spinal cord (see text footnote 1) (Milich et al., 2021), we detected changes in the expression of Tuj1 (Tubb3) in OPCs after SCI (Figures 5A, B). We found that oligodendrocyte cell lines along with neurons were Tuj1-specific expressing cells under normal conditions (Figure 5C), as we found *in vitro* (Figures 3A–D). In particular, the expression of Tuj1 in OPCs increased gradually at 1 to 3 days post injury (dpi) after SCI but decreased at 7 dpi. Furthermore, we also found that most of neuron markers were expressed in OPCs [such as Map2, Mapt (Tau), Nefm (Neurofilament), Uchl1, Doublecortin (DCX), and Gap43] (Supplementary Figures 7A–E). Some of these markers were increased in the early stage after injury and then gradually decreased [such as TUBB3 (Tuj1), Smn1, Uchl1, Gap43] (Figures 5A–C and Supplementary Figure 7F). While, some markers were decreased in the early stage of injury, and then gradually recovered (such as DCX, Nefm, NeuroD, Tbr1, Thy1/Cd90, Mapt, Map2) (Figure 5D and Supplementary Figure 7F). Furthermore, when compared with adult mice, most of neuron markers were well preserved in juvenile mice (Supplementary Figure 7G). Meanwhile, the spinal cord neurons almost did not express CSPG4 (NG2) under normal conditions or after injury (Supplementary Figure 8). Therefore, these Tuj1 + cells may be partly derived from NG2 positive OPCs. These results indicate that OPCs may have the potential to *trans*-differentiate into neurons but not successfully after SCI, which may be due to unknown environmental factors or a lack of factors mediating transformation.

Recent study confirmed that SOX2-mediated *in vivo* reprogramming of NG2 + glial cells could produce new excitatory and inhibitory propriospinal neurons, reduce glial scarring, and promote functional recovery after SCI (Tai et al., 2021). The microtubule-associated protein doublecortin (DCX) is normally expressed in neuroblasts and immature neurons and can serve as a reliable marker for adult neurogenesis (Tai et al., 2021). ASCL1, a master regulator expressed in neural progenitors and critical for neuronal differentiation and adult neurogenesis, was detected in 28.6% of SCI-induced DCX + cells (Tai et al., 2021). By analyzing online scRNA-seq data from the injured mouse spinal cord (Milich et al., 2021), we found that Sox2, DCX, and ASCL1 were highly or specifically expressed in OPCs under normal conditions, while these factors were significantly down-regulated after SCI (Figure 5D). These results further confirmed that OPCs may have the potential to *trans*-differentiate into neurons but not successful after SCI.

Furthermore, through RNA-seq, we also detected the transcriptional changes in Sox2, DCX, and ASCL1 in the lesion area at different ages and time. Compared with juvenile mice, we confirmed that the transcripts of DCX and ASCL1, along with MBP, Oligo2, and Tubb3 (Tuj1), in adult mice significantly decreased at 2 WPI (Figure 5E), which indicated a large number of oligodendrocytes and neuron death in the lesion area. Meanwhile, compared with juvenile mice, transcripts of SOX2 and NG2 in adult mice were significantly up-regulated but did not promote neurogenesis (Figure 5E). We also found that the transcripts of ASCL1 gradually decreased and NG2 (CSPG4) gradually up-regulated with time after SCI (Figure 5F). The transcripts of DCX, SOX2, and Tubb3 (Tuj1) underwent a process of dynamic change, first decreasing from 1 dpi to 3 dpi but gradually increasing from 1 to 2 WPI, and then decreasing again at 8 WPI (Figure 5F). These results suggest that the injured tissue may try to promote the regeneration of neurons after SCI but failed due to unknown factors.

### 3.6 ER stress inhibition could protect OPCs from death and inhibit oligospheres differentiation

Previously, we confirmed that inhibition of endoplasmic reticulum stress (ER stress) related signal pathways can effectively improve neuronal death (Zhao et al., 2017a,b). Irreversible ER stress would trigger cell death initiated by the activation of three ER stress sensors separated from immunoglobulin heavy chain binding protein (BiP/GRP78) respectively, including inositol-requiring protein 1α (IRE1α), protein kinase RNA-like ER kinase (PERK) and activating transcription factor 6 (ATF6) (Hetz, 2012). Whether inhibiting ER stress would also protect OPCs still remains unclear.

**FIGURE 6:**
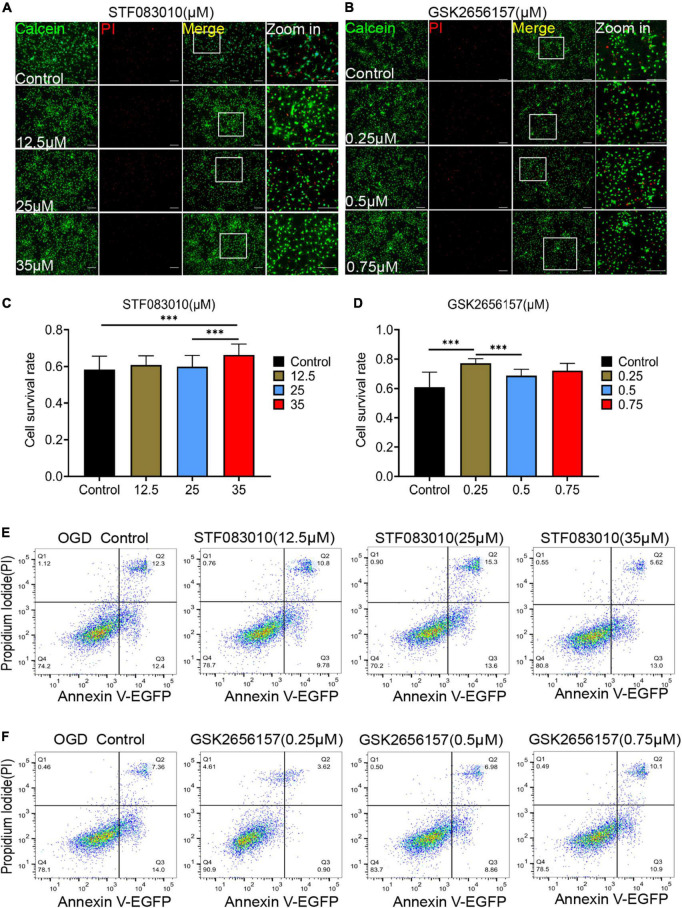
Endoplasmic reticulum stress (ER) stress inhibition protect OPCs from death after OGD stimulation. **(A,B)** The effects of different concentrations of STF083010 and GSK2656157 on OPCs mortality after OGD stimulation by Calcein AM/PI double staining. Scale bars had been indicated in pictures. *n* = 3 biological repeats. **(C,D)** The histograms revealed the statistical results of oligodendrocytes mortality after OGD stimulation. *n* = 3 biological repeats. Values are the mean ± SEM. Statistical significance was determined by one-way ANOVA followed by Student Newman–Keuls *post-hoc* test. ^***^P < 0.001. **(E,F)** Through flow cytometry of apoptosis by Annexin V-EGFP/PI double staining, the results revealed the roles of STF083010 and GSK2656157 in the death of OPCs. *n* = 3 biological repeats.

Then, we tried to detected the effect of ER stress inhibition on OPCs death *in vitro* under hypoxic conditions. The different concentrations of ER stress receptors IRE1α and PERK inhibitors were used to intervene oligodendrocytes after OGD. STF083010 is the specific inhibitor of IRE1α Rnase, whereas GSK2656157 is a specific inhibitor of the PERK kinase (Zhao et al., 2017a). Firstly, we detected the effects of different concentrations of STF083010 and GSK2656157 on oligodendrocyte mortality after OGD stimulation by Calcein AM/PI double staining (Figures 6A, B). We found that 35 μM STF083010 could effectively reduce the death of OPCs (Figures 6A, C). Meanwhile, 0.25 μM GSK2656157 could significantly inhibit the death of OPCs (Figures 6B, D).

**FIGURE 7:**
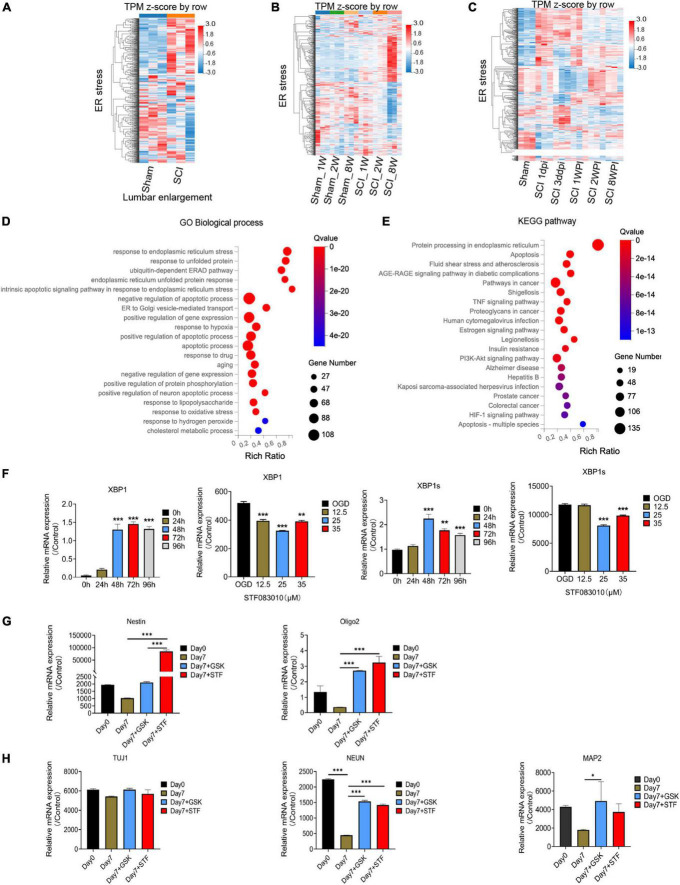
RNA-seq and bioinformatics analysis results after spinal cord injury (SCI) and RT-PCR results. **(A–C)** Heatmaps showed that the transcripts of ER stress related genes were significantly up-regulated after SCI. Dpi, day post-injury; WPI, week post-injury; Sham _1w, Sham _1 week age; Sham_2w, Sham _2 weeks age; Sham _8w, Sham_8 weeks age; SCI_1W, SCI_1 week age; SCI_2W, SCI_2 weeks age; SCI_8W, SCI_8 weeks age. **(D,E)** GO and KEGG pathway analysis also revealed that ER stress associated signaling pathways were significantly activated. **(F–H)** The RT-PCR results of Xbp1 and Xbp1s (spliced Xbp1), Nestin, Oligo2, Tuj1, NEUN, and MAP2 mRNA expression. *n* = 3 biological repeats. Values are the mean ± SEM. Statistical significance was determined by one-way ANOVA followed by Student Newman–Keuls *post-hoc* test. *P < 0.05, ^**^P < 0.01, ^***^P < 0.001.

Through flow cytometry of apoptosis by Annexin V-EGFP/PI double staining, we further confirmed that 35 μM STF083010 could effectively reduce the death of OPCs (Figure 6E), and 0.25 μM GSK2656157 could significantly inhibit the death of OPCs (Figure 6F). Through RNA-seq, we found the transcripts of ER stress related genes (Zhang et al., 2021) were significantly up-regulated after SCI (Figures 7A–C). The GO and KEGG pathway analysis also revealed that ER stress associated signaling pathways were activated (Figures 7D, E). We further screened the dynamic changes of ER stress key genes after SCI in scRNA-seq and RNA-seq data. We found that ER stress was activated in the early stage of SCI, and gradually decreased (Supplementary Figures 9A–E). Our RT-PCR results also revealed the unspliced and spliced mRNA expression of X-Box Binding Protein 1 (Xbp1), as the downstream factors of ER stress, were gradually increased after OGD stimulation, which could be significantly inhibited by STF083010 treatment (Figure 7F). Thus, inhibiting ER stress could reduce OPCs death under hypoxic conditions.

The ER machinery integrates various intracellular and extracellular signals. Previous study found that ER stress regulated the intestinal stem cell state through monoclonal antibody to C-Terminal binding protein 2 (CTBP2), and showed CtBP2 mediates ER stress-induced loss of stemness (Meijer et al., 2021). Therefore, we wondered whether inhibition of ER stress would have effect on oligospheres differentiation. Interesting, we found that inhibition of ER stress during oligospheres differentiation could significantly increase the expression of stem cell marker Nestin (Figure 7G). Furthermore, we found that mRNA expression of oligodendrocyte lineage cells marker Oligo2, and neuron markers NEUN and MAP2 were significantly increased after ER inhibition (Figure 7H). Thus, ER inhibition may be associated with stemness maintenance and regulating the differentiation of oligospheres.

## 4 Discussion

There were several findings in this study: (1) OPCs differentiation and oligodendrocyte morphology were significantly different between brain and spinal cord; (2) OPCs were involved in oligospheres formation, which further differentiated into neuron-like cells. (3) A majority of Tuj1 + MBP + NG2 + triple-positive cells with neurite-like branches were detected during the differentiation of oligospheres; (4) OPCs were significantly activated in the lesion area and lumbar enlargement after SCI; (5) Combining scRNA-seq and RNA-seq data, the neurogenesis potential of OPCs were detected after SCI; (6) inhibition of ER stress could effectively attenuate OPCs death; (7) ER inhibition may regulate the stemness and differentiation of oligospheres.

Oligodendrocyte progenitor cells are the source for new mature oligodendrocytes during the regeneration of damaged myelin (Zawadzka et al., 2010; Gibson et al., 2014; McKenzie et al., 2014; Hughes et al., 2018). We found that the OPCs differentiation and oligodendrocyte morphology of brain and spinal cord were significantly different. It has been established that oligodendrocytes and OPCs from different regions of the CNS have distinct origins and are functionally dissimilar, which has implications for myelination and regenerative capacity (Rowitch and Kriegstein, 2010; Bechler et al., 2015). OPCs from the cortex and spinal cord produce myelin sheaths of different lengths when cultured under similar conditions (Bechler et al., 2015), and transplanted OPCs from white matter differentiate faster than OPCs from gray matter (Viganò et al., 2013). Cholesterol is an integral component of the myelin sheath. A recent study demonstrated that OPCs in the spinal cord exhibit higher levels of cell-autonomous cholesterol biosynthesis than the equivalent stage precursors in the brain (Khandker et al., 2022). Conversely, brain oligodendrocytes have a higher capacity for extracellular cholesterol uptake (Khandker et al., 2022). In addition, OPCs in different regions show different responsiveness to growth factors (Hill et al., 2013) and vary in their capacity to differentiate when transplanted into other CNS areas (Viganò et al., 2013). Furthermore, the physiological properties of OPCs have been found to diversify increasingly over time (Spitzer et al., 2019), and mature oligodendrocytes also show transcriptional heterogeneity (Floriddia et al., 2020). Thus, oligodendrocyte heterogeneity should be taken into consideration in future OPCs transplantation to promote myelin regeneration after SCI. Previously, several studies have described that OPCs can form oligospheres (Avellana-Adalid et al., 1996; Chen et al., 2007; Pedraza et al., 2008; Gibney and McDermott, 2009; Li et al., 2015). Importantly, oligospheres-derived OPCs preserve the capacity to express differentiated antigenic and metabolic phenotypes (Avellana-Adalid et al., 1996). A previous study showed that the B104CM-containing oligospheres medium was most effective in inducing oligospheres formation from neurospheres (Chen et al., 2007). Meanwhile, the efficiency of forming oligospheres was low when using the neonatal brain compared to the embryonic brain (Chen et al., 2007). They also reported that no neuronal cells, as indicated by the expression of Tuj1, were detected in the isolated OPCs population (Chen et al., 2007). In our study, oligospheres were also effectively generated from the neonatal brain in our culture system. Interestingly, we firstly detected the intermediate states of oligodendrocytes and neurons during oligospheres differentiation, for a number of Tuj1 + MBP + NG2 + triple-positive OPCs on the sixth day after plating were observed, among which a number of these cells had Tuj1-positive neurite-like branches. Indeed, we found that MBP + positive cells were gradually decreasing in the process of oligospheres culture and differentiation. We did not add other factors to the medium to promote the differentiation of neurons. The OPCs medium is refer to the previous reports (Hattori et al., 2017; Miyamoto et al., 2020). The OPCs medium is considered to be used for OPCs proliferation, but not for OPCs differentiation. Oligospheres were formed by OPCs cells in suspension culture, and then gradually differentiated into neuron like cells after PDL coating and adherent culture. We speculated that OPCs may acquire part of neural stemness during the process of oligospheres formation, and finally differentiate into neuron like cells. Thus, we identify a previously undescribed findings that OPCs formed oligospheres could differentiate into neuron-like cells. Furthermore, we also confirmed the significant activation and neurogenesis potential of OPCs after SCI. These findings highlighted the neurogenesis potential of OPCs, which may provide a new insight for spinal cord repair.

Directly converting non-neuronal cells into induced neurons has emerged as an innovative strategy for brain and spinal cord repair. OPCs have attracted extensive attention because of their potential to self-renew, differentiate and repair the myelin sheath. Recent studies reported that overexpression of a variety of factors can induce NG2 cells (mainly OPCs) to *trans*-differentiate into neurons, such as by Sox2 and adeno-associated virus (AAV)-based reporter system (Heinrich et al., 2014; Torper et al., 2015; Tai et al., 2021). Recently, Tai et al. (2021) found that ectopic SOX2-induced neurogenesis proceeding through an expandable ASCL1 + progenitor stage was necessary and sufficient to reprogram NG2 glia (mainly OPCs) into neurons after SCI. In addition, they also demonstrated that NG2 glia were the main source of ASCL1 + cells (Tai et al., 2021). These findings indicate that OPCs have the latent potential for neurogenic regeneration, which may represent a potential target for reprogramming strategies for spinal cord repair. OPCs may be one of the main reservoirs that could further generate neurons after SCI. Thus, the rational use of transgenic or virus transfection methods and biomaterials may promote the transformation of OPCs into neurons in injured spinal cord.

Age plays a key role in nerve regeneration and age-dependent OPCs heterogeneity should be considered (Baldassarro et al., 2019, 2020). In this study, the primary cells were extracted from the brain tissue of newborn rats (P1-2), so we did not compare the OPCs between juvenile and adult. According to our experience, it may be difficult to extract primary cells from adult spinal cord and brain, because most cells are difficult to survive during the extraction. Additionally, we established SCI models in juvenile (1 week old and 2 weeks old) and adult mice, and obtained transcriptome data of injured spinal cord. We found juvenile mice had strong repair ability after SCI, and the injured area can be effectively repaired along with better functional prognosis than that of adult mice (unpublished data). Furthermore, when compared with adult mice, our RNA-seq results also showed that the neuron related markers in juvenile mice were well preserved after SCI. Therefore, juvenile mice may have stronger nerve regeneration ability than adult mice. However, there is still a lack of research on the neurogenesis potential of OPCs in juvenile mice. More work is needed in the future.

Endoplasmic reticulum stress refers to the process that unfolded or misfolded proteins accumulate in the ER and activate the unfolded protein response (UPR) under various pathological conditions, so as to reduce unfolded protein load and restore ER homeostasis (Hetz, 2012). However, irreversible ER stress would trigger cell death initiated by the activation of three ER stress sensors separated from immunoglobulin heavy chain binding protein (BiP/GRP78) respectively, including IRE1α, PERK and activating transcription factor 6 (ATF6) (Hetz, 2012). Previously, we have confirmed that inhibition of ER stress related signal pathways can effectively improve neuronal death (Zhao et al., 2017a,b). In this study, we also confirmed that inhibiting ER stress could reduce OPCs death under hypoxic conditions. In contrast, a recent study also found that the IRE1-XBP1-mediated UPR signaling pathway contributes to restoration of ER homeostasis in oligodendrocytes and is necessary for enhanced white matter sparing and functional recovery post-SCI (Saraswat Ohri et al., 2021). Oligodendrocyte-specific deletion of Xbp1 exacerbates the ER stress response and restricts locomotor recovery after thoracic SCI (Saraswat Ohri et al., 2021). Thus, there were inconsistent association between oligodendrocyte death and ER stress, mainly for the double-sword role of ER stress in regulating programmed cell death and homeostasis to protect tissue integrity.

Several studies confirmed that ER plays an important role in regulating stem cells differentiation. Liu and colleges (Liu et al., 2012) confirmed that human embryonic stem cells (hESCs) cell line H9 express high levels of UPR markers, such as XBP1 and p-eIF2α (a downstream factor of ER Stress) that are substantially down-regulated in differentiated cells. Furthermore, researchers found that inhibition of ER stress supported self-renewal of mouse ESCs (mESCs) (Chen et al., 2014; Kratochvílová et al., 2016). Another study also found that ER stress regulates the intestinal stem cell state (Meijer et al., 2021). Additionally, adaptive ER stress signaling *via* IRE1α-XBP1 preserves self-renewal of hematopoietic and pre-leukemic stem cells (Liu et al., 2019). We also identified that ER may be associated with differentiation and stemness of oligospheres. While, more works are needed to explore the mechanisms.

There were also some limitations in this study. To our knowledge, there are no effective methods to isolate 100% pure oligodendrocytes to date, partly for current markers can also be expressed in neural stem cells. However, we did observe the intermediate states of oligodendrocytes and neurons during oligospheres differentiation, which further highlighted the neurogenesis potential of OPCs. Meanwhile, the mechanisms by which oligospheres differentiate into neuron-like cells are still unknown. Thus, further works are needed, such as lineage tracing of the generation and differentiation of oligospheres along with more in-depth characterization of neurons arise from them. Furthermore, oligospheres with biomaterials and factor modification should be transplanted into the injured spinal cord to detect their potential for neurogenesis and repair.

## Data availability statement

The datasets presented in this study can be found in online repositories. The names of the repository/repositories and accession number(s) can be found in the article/Supplementary material.

## Ethics statement

This animal study was reviewed and approved by Institutional Animal Care and Use Committee (IACUC) of the Tongji University School of Medicine.

## Author contributions

QZ designed and performed experiments, analyzed the data, and drafted the manuscript. YZ, YR, SY, LY, RH, SS, and XH participated in the *in vitro* and *in vivo* experiment and data analysis. NX, LC, and RZ conceived and designed the experiments, supervised the overall project, and revised the manuscript. All authors have read and approved the article.

## Footnotes

1
https://jaeleelab.shinyapps.io/sci_singlecell/

2
https://www.ncbi.nlm.nih.gov/tools/primer-blast/index.cgi
