# Supplementary material for: Neurogenesis potential of oligodendrocyte precursor cells from oligospheres and injured spinal cord
Source: Front Cell Neurosci. 2022 Dec 22;16:1049562. doi: 10.3389/fncel.2022.1049562 (PMC9813964; doi:10.3389/fncel.2022.1049562)
Supplement: Supplementary file 1 [file Data_Sheet_1.pdf]

Supplementary Material

1 Supplementary Figures

1.1 Supplementary Figure 1

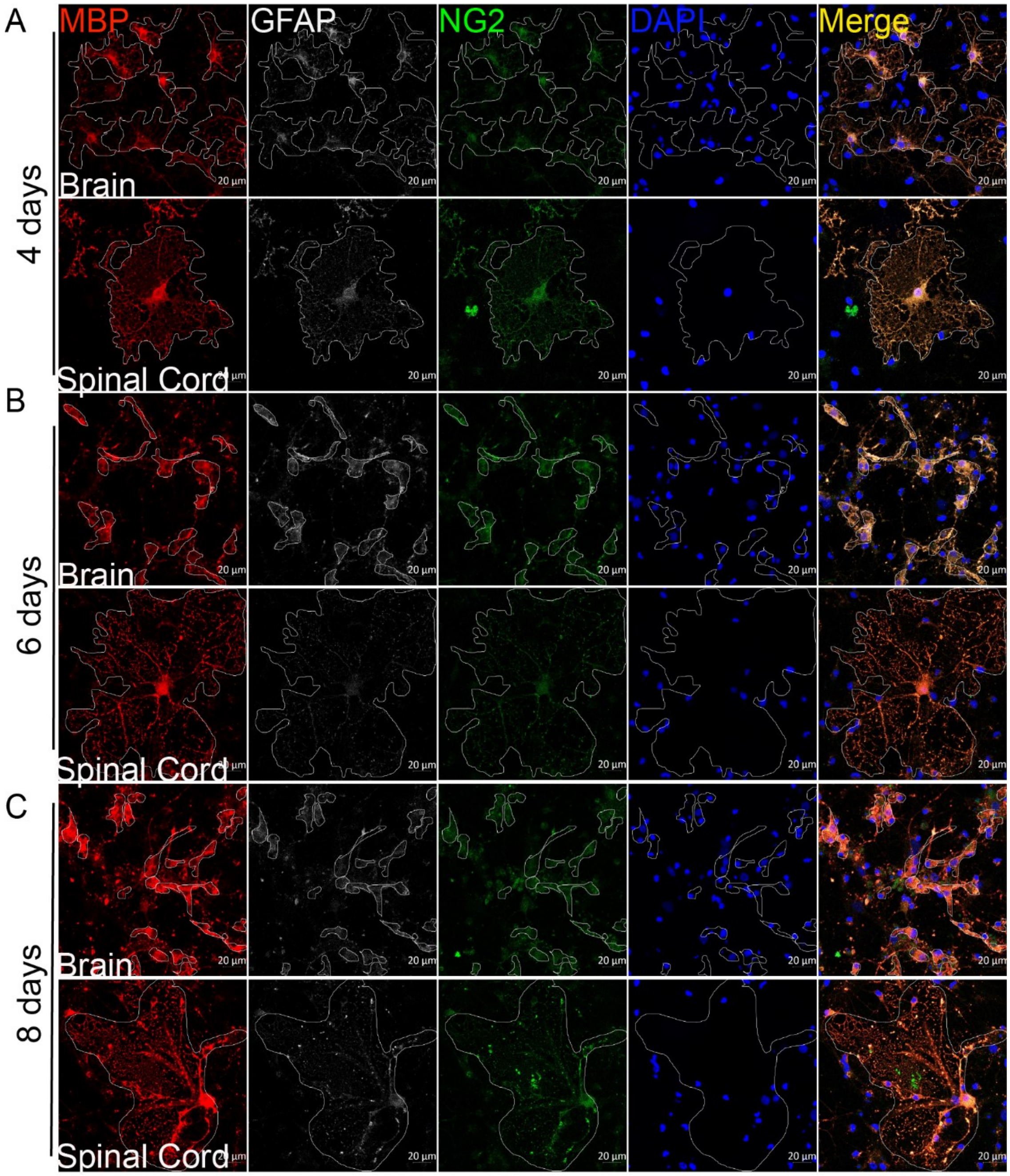

Supplementary Figure 1. The OPCs differentiation and oligodendrocytes morphology are different between the brain and spinal cord. (A-C) Zen 2.3 (Blue Edition, Carl Zeiss) software delineated the

cell morphology of oligodendrocytes. NG2 (green), MBP (red) and GFAP (white). n = 3 biological repeats. Scale bars are indicated in the pictures.

1.2 Supplementary Figure 2

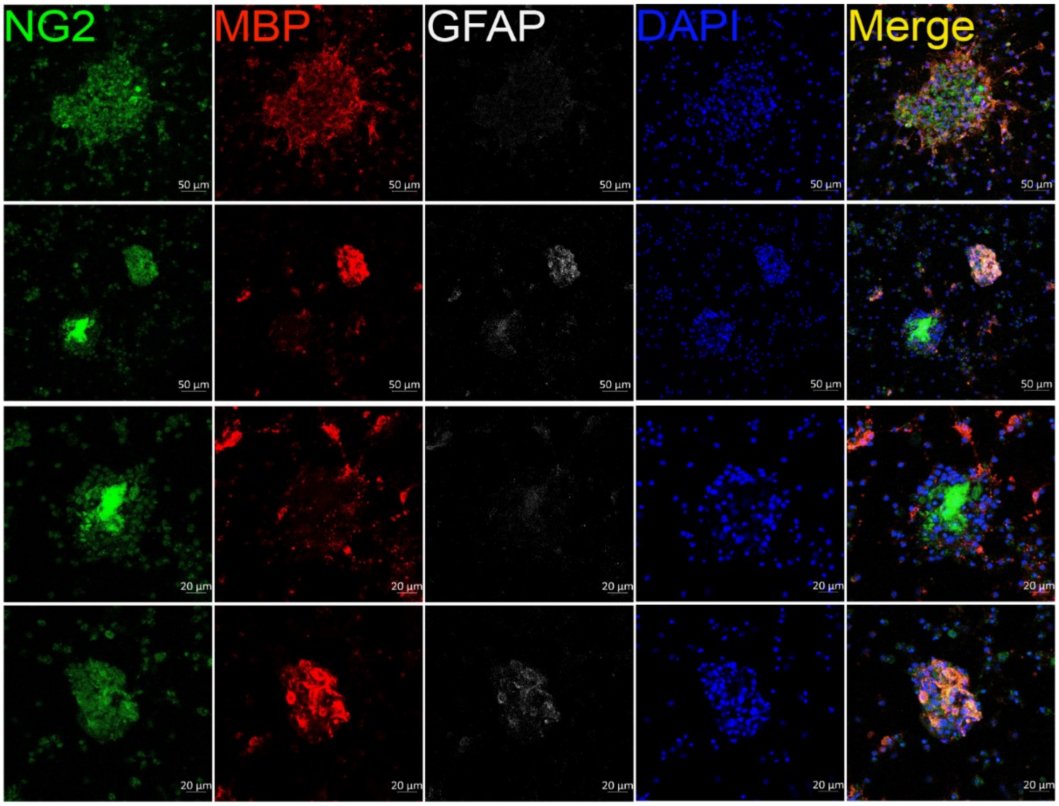

Supplementary Figure 2. OPCs are involved in oligospheres formation in vitro. Oligodendrocytes from spinal cord formed into oligospheres on the 8th day, as observed by NG2 (green), MBP (red) and GFAP (white). n = 3 biological repeats. Scale bars are indicated in the pictures.

1.3 Supplementary Figure 3

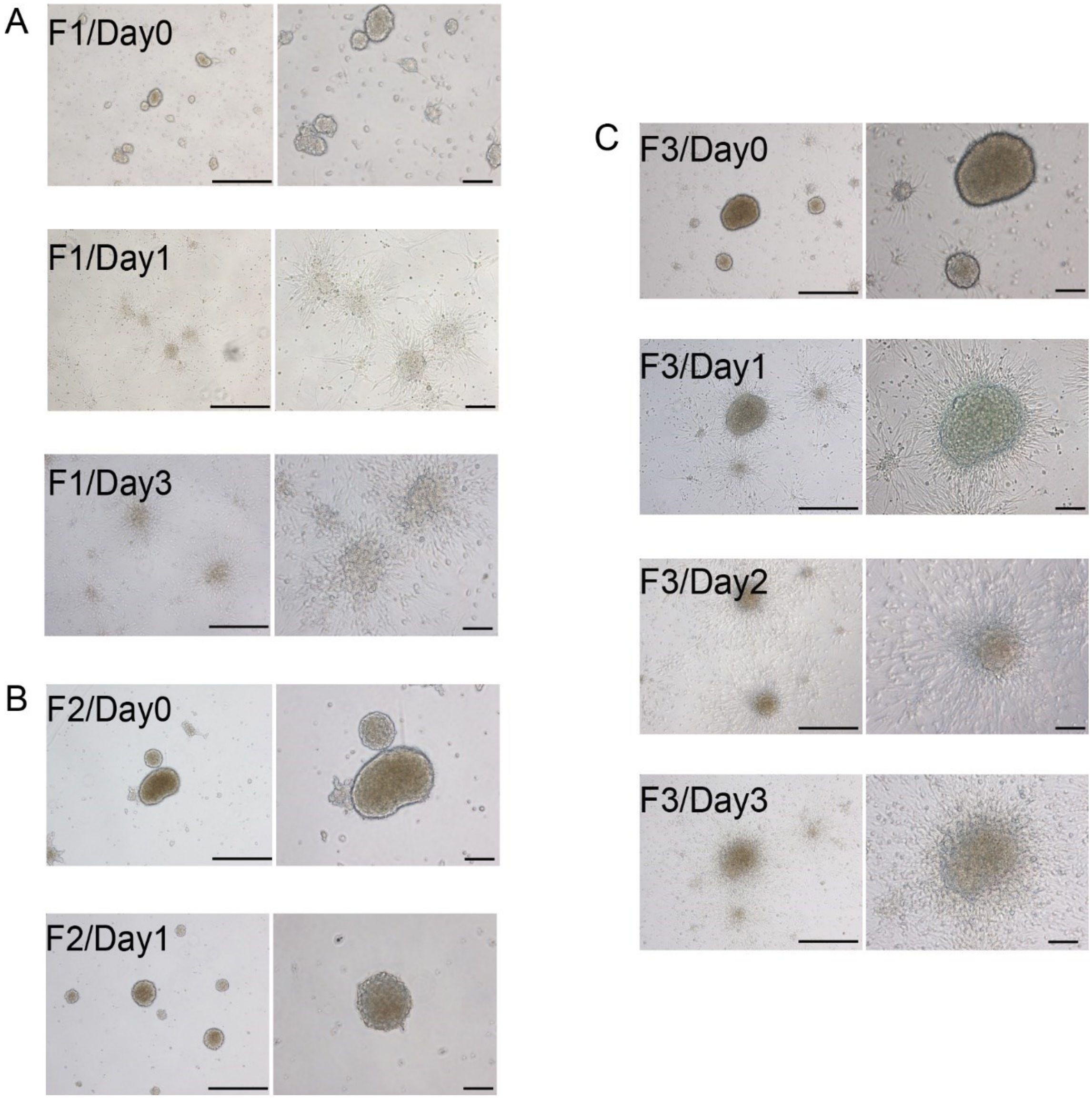

Supplementary Figure 3. OPCs are involved in oligospheres formation in vitro. (A-C) Oligospheres from different generations were plated on PDL-coated 12-well plates to observe differentiation at different time. F1= the first generation, F2= the second generation, F3= the third generation. n = 3 biological repeats. Scale bars are indicated in the pictures.

1.4 Supplementary Figure 4

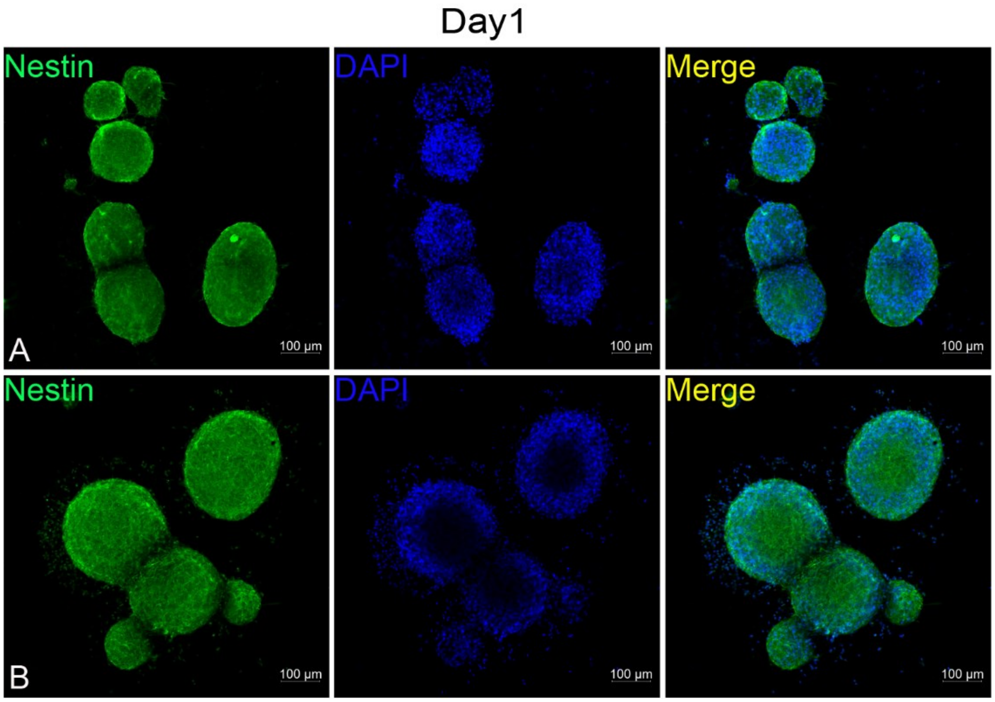

Supplementary Figure 4. Immunofluorescence staining to detect the Nestin protein expression in oligospheres. (A-B) Almost all oligospheres were Nestin positive. n = 3 biological repeats. Scale bars are indicated in the pictures.

1.5 Supplementary Figure 5

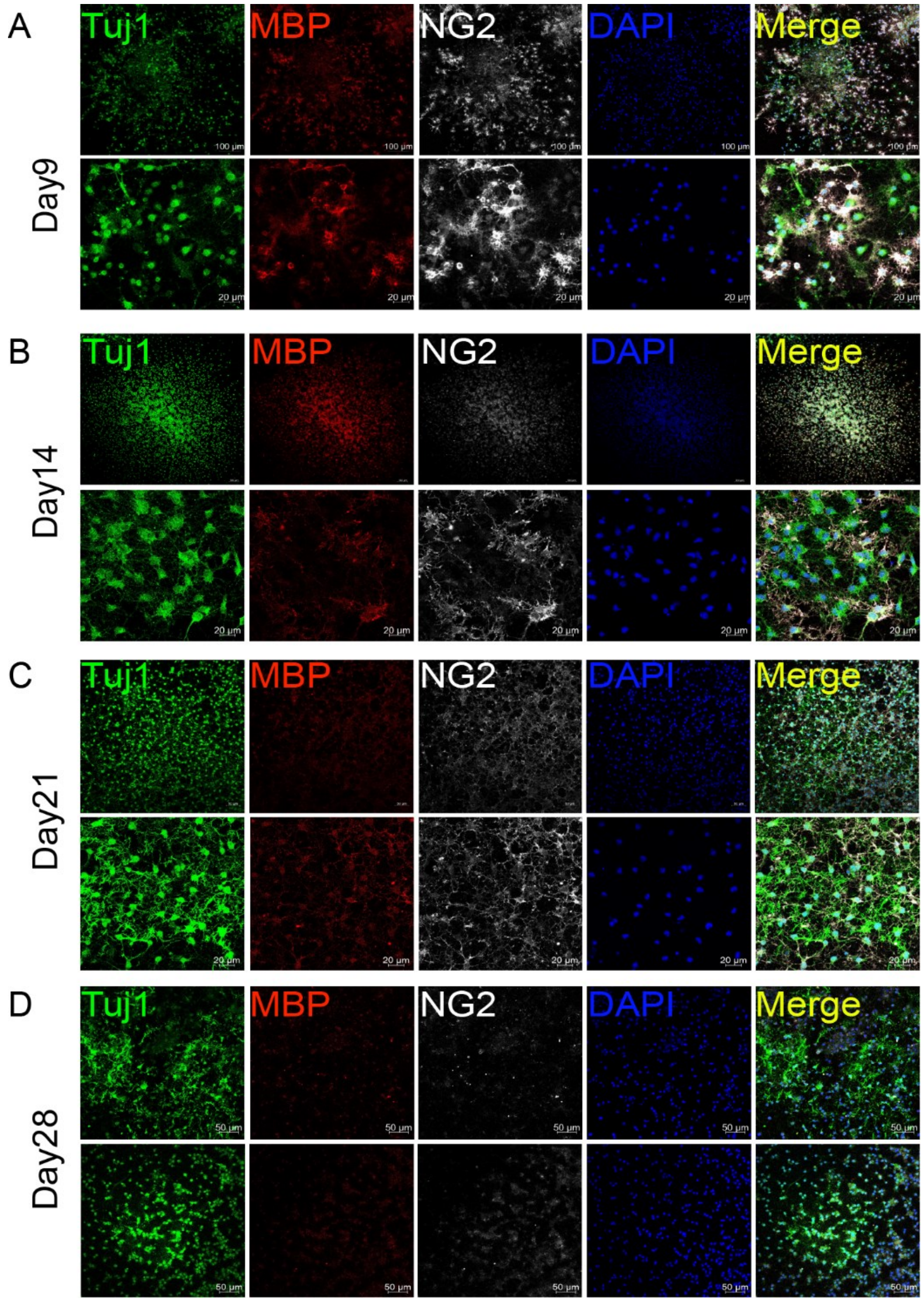

Supplementary Figure 5. Immunofluorescence staining to detect the cellular composition of oligospheres at different differentiation period. (A-D) F4 generation oligospheres were used for immunofluorescence staining to detect the cellular composition of oligospheres at different time with Tuj1 (green), MBP (red) and NG2 (white). n = 3 biological repeats. Scale bars are indicated in the pictures.

1.6 Supplementary Figure 6

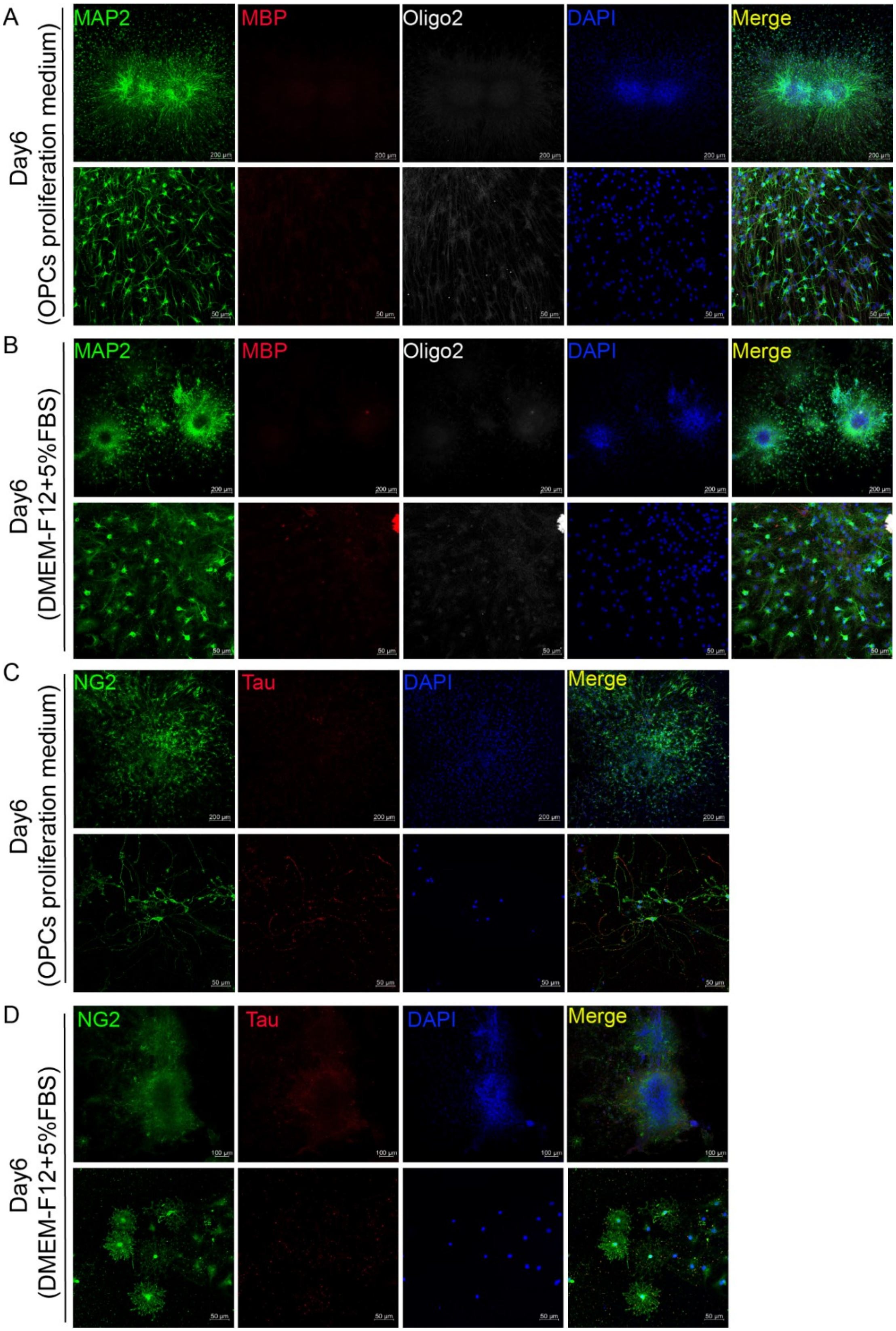

Supplementary Figure 6. Immunofluorescence staining to detect the cellular composition of oligospheres on the 6th day of differentiation with different culture mediums. (A-D) F4 generation oligospheres were used for immunofluorescence staining to detect the cellular composition of oligospheres at different time with MAP2 (green), MBP (red), Oligo2 (white), NG2 (green) and Tau (red). n = 3 biological repeats. Scale bars are indicated in the pictures.

1.7 Supplementary Figure 7

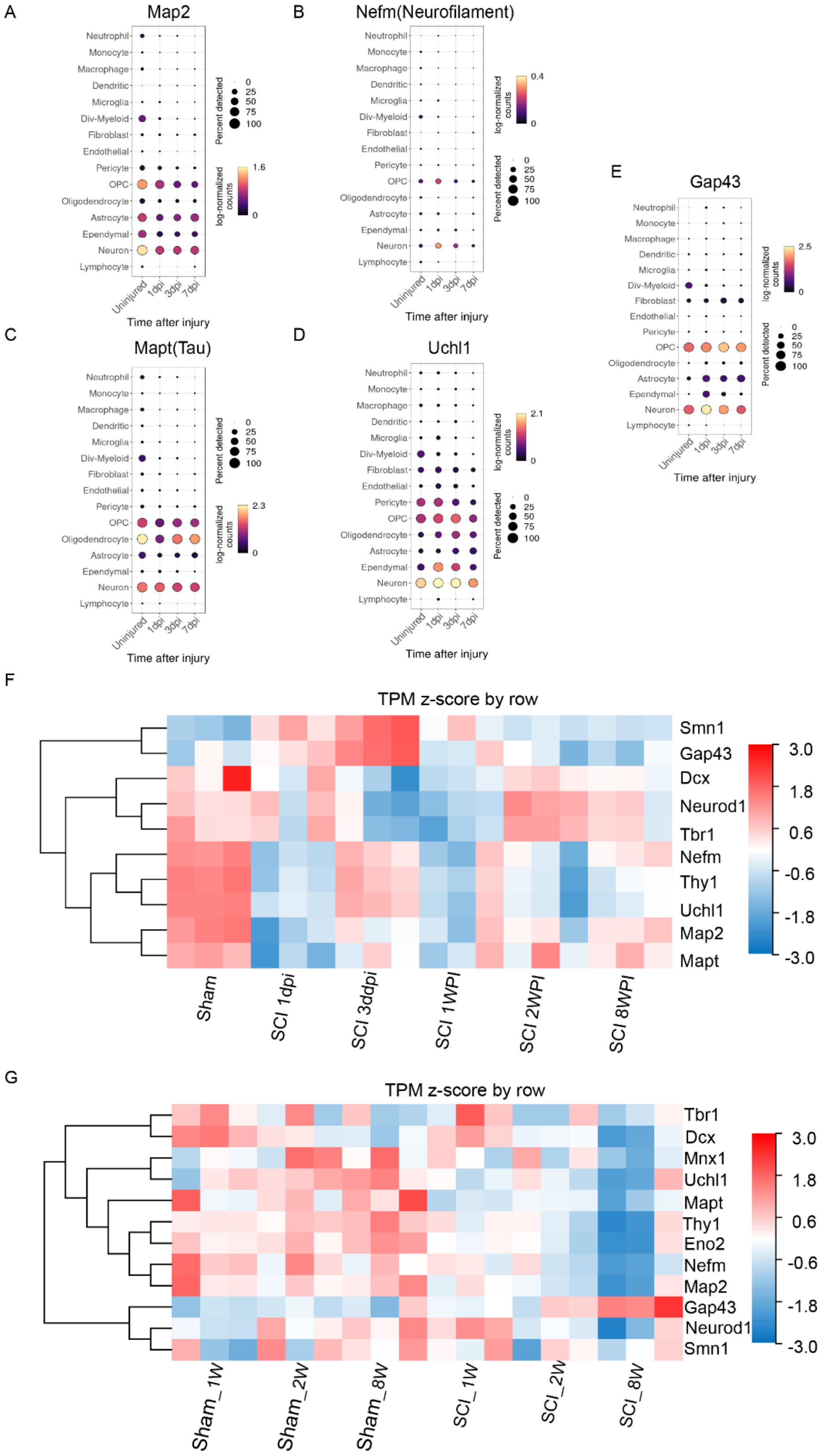

Supplementary Figure 7. Combining the scRNA-seq and RNA-seq data to investigate the neurogenesis potential of OPCs after SCI. (A-E) scRNA-seq results show that most of neuron markers were expressed and undergoing dynamic changes in OPCs (such as Map2, Mapt (Tau), Nefm (Neurofilament), Uchl1, Doublecortin (DCX), and Gap43) under normal conditions and after SCI. dpi=day post-injury. (F-G) RNA-seq results showed the transcript changes of neuron markers were in a trend of dynamic change after SCI at different ages and time. n = 3 biological repeats. dpi=day post injury, WPI= week post injury, 1 W =1-week-old, 2 W =2-week-old, 8 W =8-week-old, sham_1 W= 1-week-old sham group, sham_2 W= 2-week-old sham group, sham_8 W= 8-week-old sham group, SCI_1 W= 1-week-old SCI group, SCI_2 W= 2-week-old SCI group, SCI_8 W= 8-week-old SCI group.

1.8 Supplementary Figure 8

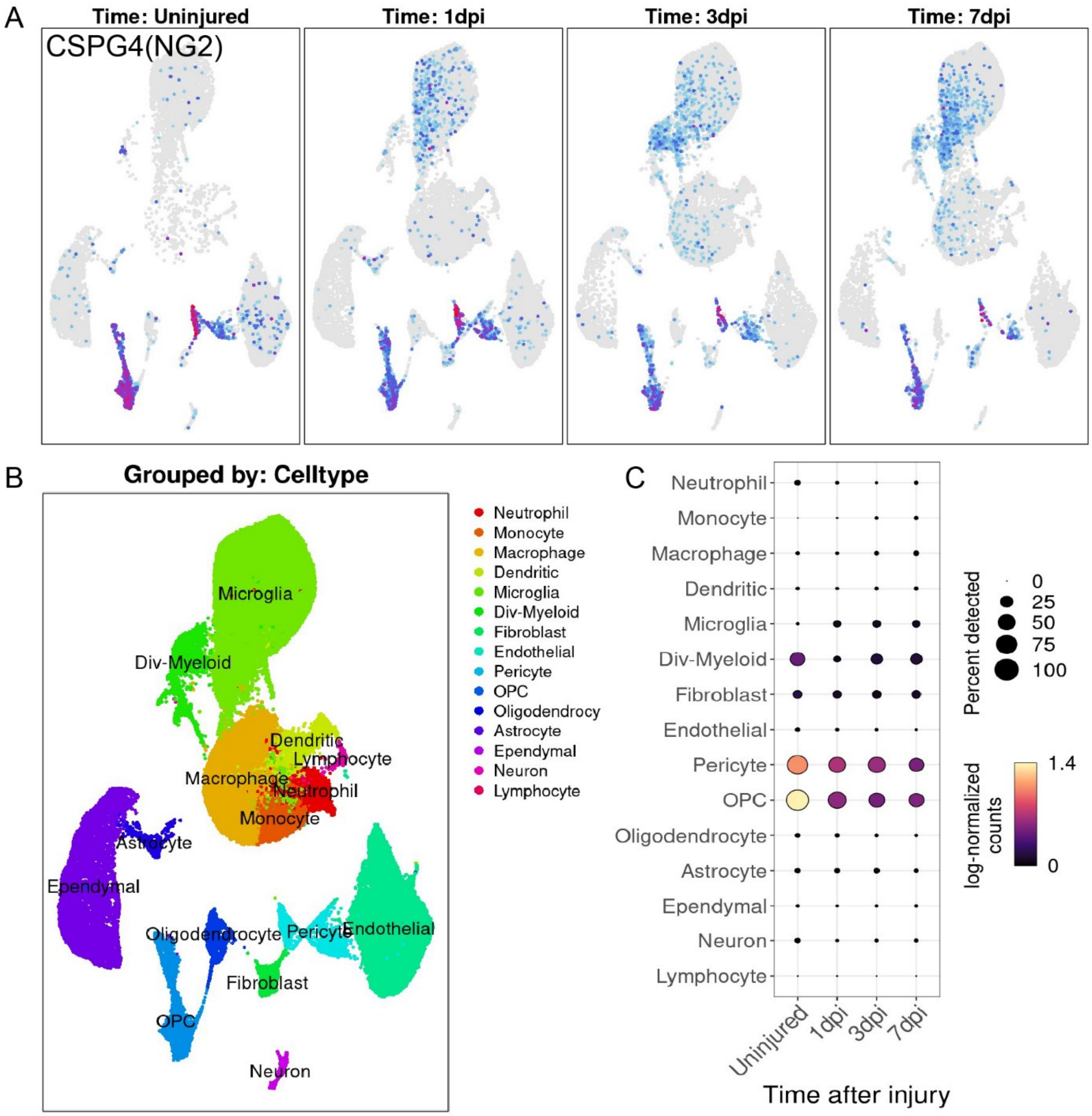

Supplementary Figure 8. The CSPG4 (NG2) expression in scRNA-seq data. (A-C) scRNA-seq results showed that the spinal cord neurons almost did not express CSPG4 (NG2) under normal conditions or after SCI. dpi=day post injury.

1.9 Supplementary Figure 9

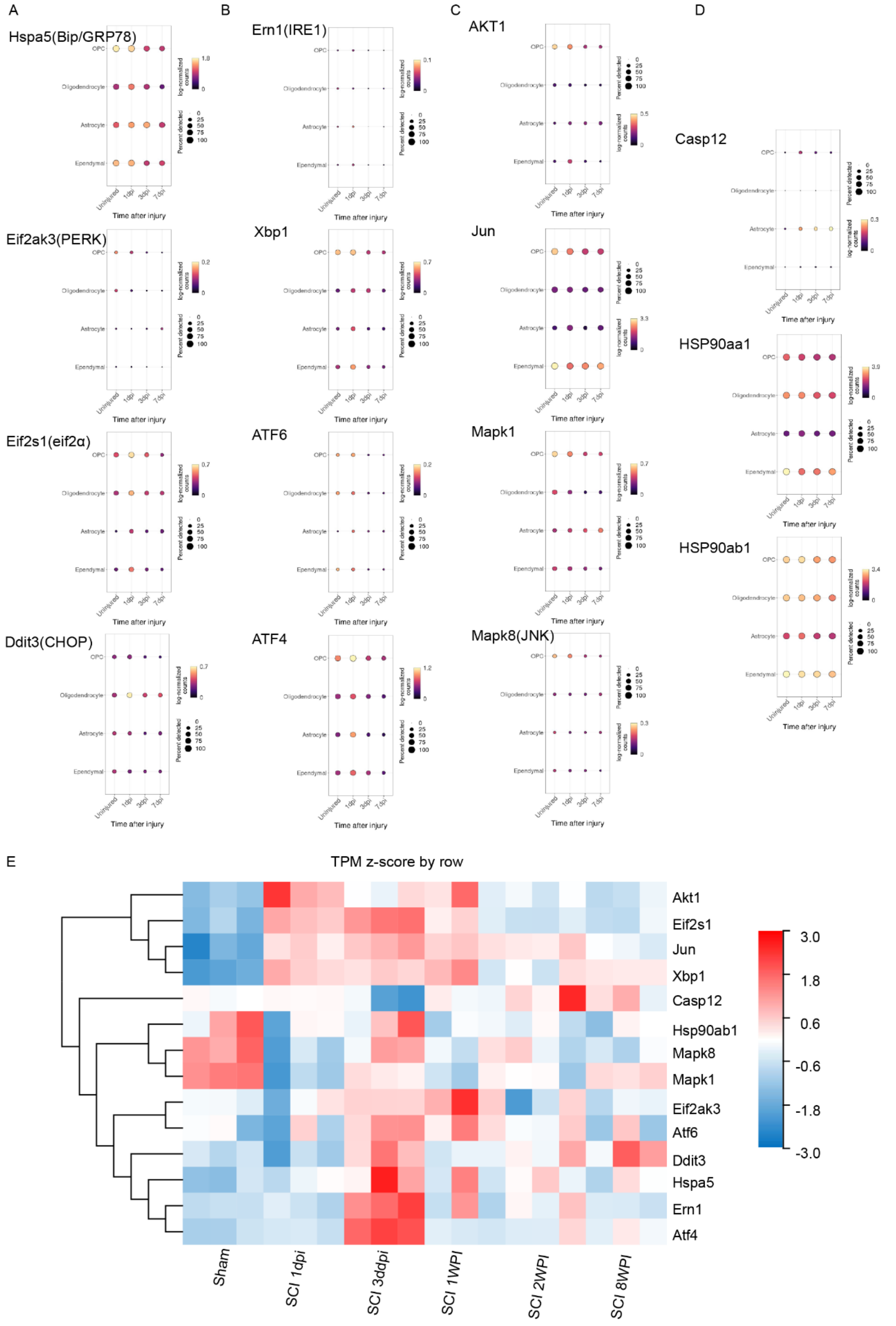

Supplementary Figure 9. Combining the scRNA-seq and RNA-seq data to investigate the dynamic changes of ER stress key genes after SCI. (A-E) scRNA-seq and RNA-seq data showed that the transcript changes of ER stress associated key genes were increased in the early stage of SCI, and gradually decreased at different time. n = 3 biological repeats. dpi=day post injury, WPI= week post injury.

2 Supplementary Table

Supplementary Table1.Primers for RT-PCR

Primer name	Primers (5'to3')	
Xbp1	forward	*CACTCAGACTACGTGCG*
	reverse	*GAGTTCCTCCAGATTAGCAG*
Xbp1s	forward	*TAGAAAGAAAGCCCGGATGA*
	reverse	*TCTCAATCACAAGCCCATGA*
NESTIN	forward	*GTGACCCTTGGGTTAGAGGC*
	reverse	*CTGGCAAAATGCCTTGGGTC*
TUJ1	forward	*GGCAACTATGTGGGGGACTC*
	reverse	*GCACCACTCTGACCGAAGATA*
NeuN	forward	*GACCAATAAGAAGCCTGGGAAC*
	reverse	*TCCGTAAGTTGGAATGGGGG*
Olig2	forward	*CCTGCTCAAGTCTCCATCGG*
	reverse	*CCCCTTCTGGCAACAGAGTC*
Map2	forward	*CCAGAACATACCACCAGCCC*
	reverse	*CACGGGCATTTCGATGAACC*
GAPDH	forward	*AGTGCCAGCCTCGTCTCATA*
	reverse	*TGAACTTGCCGTGGGTAGAG*
